# MRI Extraprostatic Extension Grade: Accuracy and Clinical Incremental Value in the Assessment of Extraprostatic Cancer

**DOI:** 10.1155/2022/3203965

**Published:** 2022-08-30

**Authors:** Jun-Yi Xiang, Xiao-Shan Huang, Jian-Xia Xu, Ren-Hua Huang, Xiao-Zhong Zheng, Li-Ming Xue, Yu-Long Liu

**Affiliations:** ^1^Department of Oncology, The Second Affiliated Hospital of Soochow University, Suzhou 215004, China; ^2^Department of Radiology, The Second Affiliated Hospital of Zhejiang Chinese Medical University, Hangzhou 310005, China; ^3^Department of Radiation, Renji Hospital, School of Medicine, Shanghai Jiaotong University, Shanghai 200000, China; ^4^State Key Laboratory of Radiation Medicine and Protection, School of Radiation Medicine and Protection, Medical College of Soochow University, Suzhou 215123, China; ^5^Collaborative Innovation Center of Radiological Medicine of Jiangsu Higher Education Institutions, Suzhou 215123, China

## Abstract

**Objective:**

The purpose was to compare the accuracy of extraprostatic extension (EPE) grade on MRI predicting EPE with Partin tables, Memorial Sloan Kettering Cancer Center nomogram (MSKCCn), and combined models and to analyze the clinical incremental value of EPE grade.

**Materials and Methods:**

105 prostate cancer patients confirmed by pathology after radical prostatectomy in our hospital from 2017 to 2021 were selected. The clinical stage, PSA, Gleason score, number of positive biopsy cores, and percentage of positive biopsy cores were recorded. Evaluate EPE grade according to EPE grade criteria, and calculate the probability of predicting EPE with Partin tables and MSKCCn. EPE grade is combined with Partin tables and MSKCCn to construct EPE grade+Partin tables and EPE grade+MSKCCn models. Calculate the area under the curve (AUC), sensitivity, and specificity of EPE grade, Partin tables, MSKCCn, EPE grade+Partin tables, and EPE grade+MSKCCn and compare their diagnostic efficacy. The clinical decision curve was used to analyze the clinical net income of each prediction scheme.

**Results:**

The AUC of EPE grade was 0.79, Partin tables was 0.50, MSKCCn was 0.78, the EPE grade+Partin table model was 0.79, and the EPE grade+MSKCCn model was 0.83. After EPE grade was combined with Partin tables and MSKCCn, the diagnostic efficiency of clinical model was significantly improved (*P* < 0.05). There was no significant difference in the diagnostic efficacy of the combined model compared with the single EPE grade (*P* > 0.05). The calibration curve of the combined model shows that it has a good calibration degree for EPE. In the analysis of the decision curve, the net income of the EPE grade is higher than that of Partin tables and MSKCCn and is equal to the EPE grade+Partin tables and is slightly lower than that of EPE grade+MSKCCn. The clinical net income of the combined model is obviously higher than that of individual clinical models.

**Conclusion:**

The accuracy of EPE classification in predicting prostate cancer EPE is high, and combined with the clinical model, it can significantly improve the diagnostic efficiency of the clinical model and increase the clinical benefit.

## 1. Introduction

Prostate cancer (PCa) is the most common male malignancy worldwide [1], and it is also a malignant tumor with a greatly increased incidence in my country. Extraprostatic extension (EPE) is an important pathological feature of prostate cancer, which increases the incidence of positive surgical margins and biochemical recurrence and reduces overall rates after radical prostatectomy [2, 3]. A precise preoperative prediction of EPE is crucial to determining the type of treatment to be used. Prostate cancer patients without extracapsular invasion can undergo nerve-sparing radical resection. For patients with EPE, non-nerve-sparing radical resection or preoperative neoadjuvant therapy is required [4]. At present, a large number of studies are trying to find the indicators and methods of EPE that can be accurately predicted from imaging, clinical, pathological, and other aspects, in order to guide the selection of clinical treatment plans and ultimately achieve the goal of improving the long-term prognosis of patients and improving the overall survival rate of patients.

From a clinical and pathological perspective, for the preoperative evaluation of EPE, domestic and foreign scholars have proposed many clinical models, which are the common recognized Partin tables, Memorial Sloan Kettering Cancer Center (MSKCC) nomogram, and prostate cancer risk assessment (CAPRA) score. These models were constructed based on clinical and biopsy pathological indicators, including clinical T stage, prostate-specific antigen (PSA) level, biopsy Gleason score, and needle biopsy positive ratio. However, among these models, the area under the curve (AUC) ranged from 0.61 to 0.81 based on their diagnostic performance [5–8].

From an image perspective, MRI has been shown to be able to predict EPE in several studies, but the diagnostic performance and sensitivity are not high, because MRI evaluation of EPE was mainly based on the subjective judgment of multiparameter images to define positivity or negativity of EPE. Results of a meta-analysis showed that the sensitivity of MRI to assess EPE was 0.55 (95% CI 0.43-0.66) [9]. Recently, a new MRI grading system for predicting EPE was proposed [10], it was called EPE grade. EPE grade adds quantitative parameters to the original multiparameter imaging evaluation. When compared with ESUR score and Likert score, EPE grade has the highest correlation with histological EPE and has better diagnostic performance [11]. The main advantage of EPE grade system is its simplicity, which does not incorporate complex imaging features.

Therefore, the aim of this study was to compare the accuracy of EPE grade, Partin tables, MSKCCn, and combined models in predicting EPE while analyzing the clinical incremental value of EPE grade.

## 2. Materials and Methods

### 2.1. Clinical Data

A total of 105 prostate cancer patients with pathological confirmation in our hospital from 2017 to 2021 and diagnosed with T ≤ T2 stage on digital rectal examination (DRE) were enrolled. Inclusion criteria were as follows: (1) digital rectal examination and T ≤ T2 stage; (2) mpMRI examination within 3 months before needle biopsy; (3) transrectal ultrasound-guided systematic prostate biopsy after imaging examination; and (4) laparoscopic radical prostatectomy. Exclusion criteria were as follows: (1) underwent transurethral resection of the prostate before surgery and (2) received androgen deprivation therapy or radiotherapy before surgery, etc. All patients' age, preoperative PSA value, digital rectal examination, and clinical stage judged by digital rectal examination were recorded. Gleason score of transrectal ultrasound biopsy and radical prostatectomy specimens was recorded. Patients and their families signed an informed consent after approval by the hospital ethics committee.

### 2.2. Imaging Examination

All patients underwent prostate mpMR examination with 1.5 T Siemens magnetic resonance system and obtained T1WI, T2WI, DWI, and DCE images. The images of all patients were evaluated by a double-blind method by two senior radiologists in the genitourinary professional group with reference to the grading criteria for extracapsular invasion of prostate cancer proposed by Mehralivand et al. [10]. The specific criteria are as follows: Grade 0, without any imaging signs of pathological EPE; Grade 1, the contact length of the curved surface reaches 1.5 cm or the capsule is raised and irregular; Grade 2, the above two features are present at the same time; and Grade 3, obvious capsule breakthrough. Our assessment results are EPE positive for all grades 1-3 and EPE negative for grade 0.

### 2.3. Pathological Examination

All patients underwent ultrasound-guided systematic biopsy of the prostate for pathology, and pathological specimens after radical resection were read by 2 senior pathologists. The number of positive biopsy cores, percentage of positive biopsy cores, and the Gleason score of biopsy were recorded. And the presence or absence of extracapsular invasion after radical resection was recorded.

### 2.4. Clinical Model

The 2012 version of the Partin tables and the MSKCCn were used to predict EPE by the above clinical and pathological data, and the predicted probability was recorded (the URL link of the Partin tables and the MSKCCn is as follows: https://www.hopkinsmedic/prostate_cancer/risk_assessment_tools/partin-tables.htmline.org/brady-urology-institute/conditions_and_treatments/prostate_cancer/risk_assessment_tools/partin-tables.html; https://www.mskcc.org/nomograms/prostate/preop;).

### 2.5. Statistical Methods

SPSS 25.0 statistical software was used for analysis; characteristic curve (receiver operating characteristic curve, ROC) was drawn for EPE grade, Partin tables, MSKCCn, and combined models, comparing the diagnostic performance of each prediction scheme through the AUC value of the area under the curve. The MedCalc statistical software Delong test was used to test the differences between the prediction schemes, and *P* < 0.05 was considered statistically significant. The Hosmer-Lemeshow goodness-of-fit test was used for the joint model to test the calibration degree of the constructed model. *P* > 0.05 was indicated as a good degree of calibration. The statistical software stata15 was used to draw the decision analysis curve of EPE for different prediction schemes, by comparing the relative positions of each curve and the net profit rate corresponding to different risk thresholds (the incidence of EPE) and analysis the clinical benefits of EPE predicted by each scheme.

## 3. Result

### 3.1. General Clinical Information

**Figure 1:**
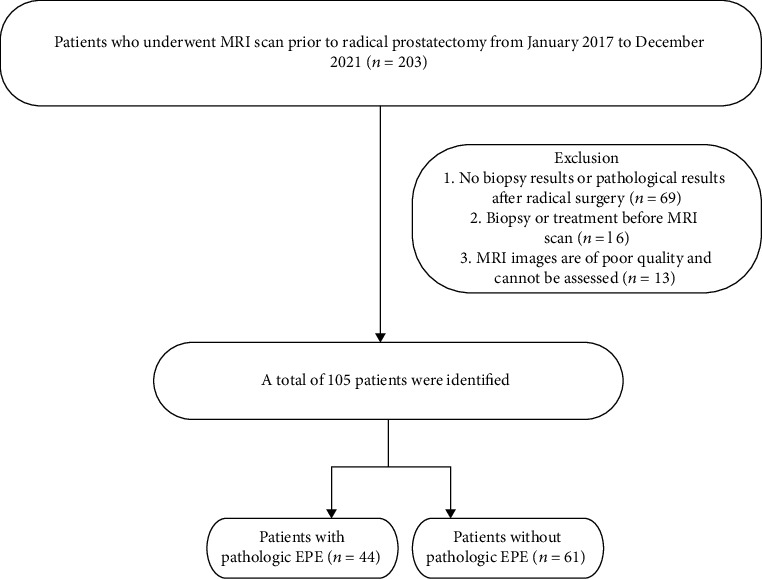
Flow diagram of participants.

The study included 105 cases (Figure 1) with an average age of 69.2 ± 5.8 years. The clinical staging by digital rectal examination is as follows: T1 76 cases, T2a 7 cases, and T2b/c 22 cases; mean PSA level (23.4 ± 24.0) mg/ml; Gleason score (3 + 3 = 6 scores, 13cases; 3 + 4 = 7 scores, 16 cases; 4 + 3 = 7 scores, 27 cases; 8 scores, 21cases; and 9-10 scores 28 cases). The average number of positive biopsy cores was 11.3 ± 1.5, and the average ratio of positive biopsy cores was 0.45 ± 0.27. Pathology after radical prostatectomy showed EPE positive in 44 cases and EPE negative in 61 cases. (Table 1).

### 3.2. The Accuracy of EPE Grade in Predicting EPE Compared with Clinical Models and Combined Models

**Figure 2:**
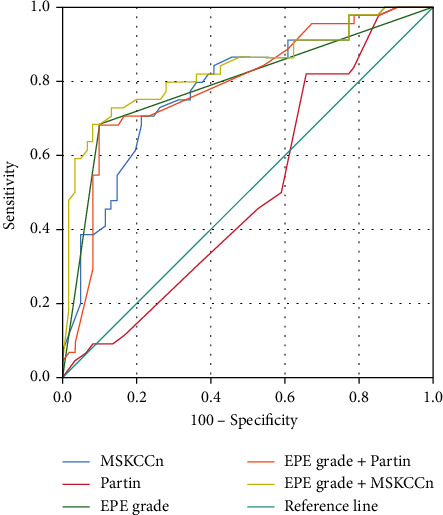
ROC curves of the EPE grade, Partin tables, MSKCCn, EPE grade+Partin tables model, and EPE grade+MSKCCn model for diagnosing EPE.

The comparison of diagnostic efficacy of EPE grade, Partin tables, MSKCCn, and combined models is shown in Tables 2 and 3 and Figure 2. The AUC value for EPE grade to diagnose EPE was 0.79 (95% CI: 0.702-0.865), the Partin table AUC was 0.50 (95% CI: 0.404 - 0.602), the MSKCCn AUC was 0.78 (95% CI: 0.690-0.856), the EPE grade+Partin table AUC was 0.79 (95% CI: 0.699-0.863), and the EPE grade+MSKCCn AUC was 0.83 (95% CI: 0.748-0.899). The diagnostic efficacy of EPE grade, MSKCCn, and combined models is comparable (AUC value comparison *P* > 0.05). Combined models have significantly higher diagnostic efficacy than Partin tables and MSKCCn (AUC value comparison *P* < 0.05), the diagnostic performance of EPE grade+MSKCCn is higher than that of EPE grade+Partin tables (*P* < 0.05), and the results show that the addition of EPE grade enhanced the diagnostic ability of clinical models. Compared with each prediction scheme, EPE grading showed moderate sensitivity and high specificity.

### 3.3. Clinical Decision Curves of the EPE Grade, Partin Tables, MSKCCn, and Combined Model

**Figure 3:**
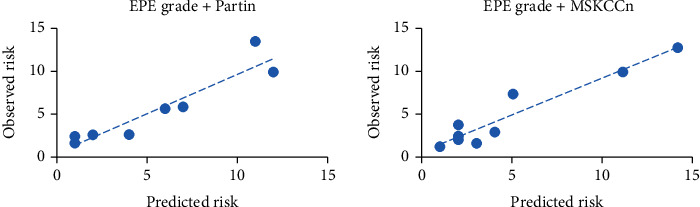
Calibration curves of the EPE grade+Partin tables model and EPE grade+MSKCCn model for evaluating EPE. Combined models were well-calibrated for EPE with nonsignificant Hosmer-Lemeshow test results (all *P* > 0.05).

**Figure 4:**
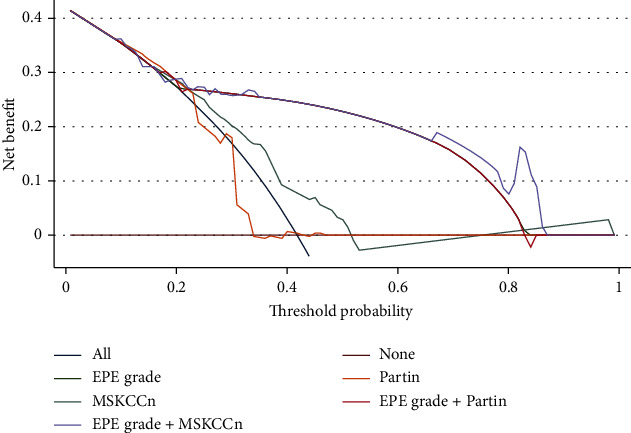
Decision curves of the EPE grade, Partin tables, MSKCCn, EPE grade+Partin tables model, and EPE grade+MSKCCn model for diagnosing EPE.

In the analysis of the clinical decision curve, it was shown that the net benefit of EPE grade was higher than that of the Partin tables and MSKCCn, was comparable with that of EPE grade + Partin tables, and was slightly lower than that of the EPE grade+MSKCCn, while the net benefit of the combined model was significantly higher than that of the individual clinical models. Finally, the EPE grade+Partin table and EPE grade+MSKCCn models were calibrated, and the calibration curve shows that the combined model has a good calibration for EPE. The statistics from the Hosmer-Lemeshow test are not significant (*P* > 0.05) (Figures 3 and 4).

## 4. Discussion

This study confirmed that the EPE rating predicts EPE well, which is higher than the Partin table prediction accuracy and comparable to the MSKCCn and combined model prediction accuracy. After combining the EPE classification with the Partin tables and the MSKCCn, the diagnostic efficacy of the clinical model in predicting EPE was significantly improved.

EPE grade is a simple and standardized grading method first proposed by Mehralivand et al. in 2019 [10] and is used for multiparametric MRI to predict pathological prostate cancer EPE. Compared with the previous MRI methods for assessing EPE (ESUR score, Likert score), EPE grade is based on only one quantitative index (surface contact length) and one qualitative index (envelope bulge, irregularity, and tumor breakthrough), and the evaluation method is simple, reproducible, and easy to teach. Based on this comparison, the EPE score and Likert score both had significant diagnostic efficacy in predicting biochemical recurrence and similar observer dependence, according to a study [12]. Park et al. [11] Compared EPE grade, ESUR score, Likert score, and surface contact length score on the basis of multiparameter MRI to predict pathological EPE, and the results of the four evaluation methods had good diagnostic efficacy (0.77-0.81, 0.79-0.81, 0.78-0.79, and 0.78-0.85, respectively). Further analysis found that EPE grade was the most correlated with pathological EPE, so EPE grade was relatively more reliable in predicting the diagnosis of pathological EPE. Subsequently, some scholars conducted external verification on the prediction of pathological EPE by EPE grade. Among them, Xu et al. [13] conducted external validation of EPE grade results, showing that EPE grade has high accuracy in predicting pathological EPE, and the sensitivity is significantly improved (sensitivity 0.767-0.778). There is a meta-analysis of EPE grade predicting pathological EPE [14], which included 4 studies and 1294 patients, and results showed that the EPE grade system showed high sensitivity (82%) and medium specificity (63%), with an AUC of 0.82 (95% CI 0.79–0.85). In our study, the diagnostic efficacy of EPE grade predictive EPE was comparable to that of the above studies, with slightly lower sensitivity and higher specificity. Compared with previous MRI assessment method, de Rooij et al. [15] performed a metasynthesis of 45 studies and 5681 patients showed that previous MRI assessment methods (excluding EPE grade) predicted EPE sensitivity of 0.57 (95% CI 0.40-0.64). At the same time, Zhang et al. [9] synthesized the meta-analysis of 17 studies and the results were similar to the above-mentioned meta-analysis, showing that the sensitivity of the previous MRI method (excluding EPE grade) to predict EPE was 0.55 (95% CI 0.43-0.66). In our study, the sensitivity of EPE grade prediction was 68%, which was significantly higher than that of previous MRI assessment methods and which had the same trend as the above study in EPE grade sensitivity and retained high specificity. The diagnostic efficacy of the EPE grade and the MSKCCn is comparable, and the diagnostic efficacy is higher than Partin tables, the clinical decision curve shows that the net benefit EPE grade under each risk threshold is higher than the Partin tables and the MSKCCn, and it can effectively improve the benefit of clinical patients. And the EPE grade provides the location of pathological EPE, which is an important information that is not found in all clinical models. Compared with clinical models alone, EPE grade can be more conducive to clinical protocol optimization.

Many studies have shown that multiparametric MRI can improve the diagnostic efficacy of clinical models. When two methods are combined, pathological EPE can be predicted more accurately. Feng et al. [8] showed that AUC values of the Partin tables and MSKCCn are 0.85 and 0.86 to predict pathological EPE alone. When MRI was added to the above clinical model, the area under the curve of the Partin tables and MSKCCn increased to 0.92 and 0.94, respectively. The study by Rayn et al. has shown that MRI predicted AUC values of 0.78 for pathological EPE, 0.66 and 0.70 for Partin tables and MSKCC line plot, and 0.80 (*P* = 0.003) and 0.80 (*P* < 0.001) after combining MRI. The study of Xiong et al. [16] enrolled 178 patients with prostate cancer. The study compared the accuracy of MRI and Partin tables, MSKCCn and CAPRA score, and their combined clinical scales to predict pathological EPE and SVI. The results showed that the AUC value of MRI predicting pathological EPE was 0.599 and showed the AUC value of 0.652, 0.763, and 0.780 after combining the Partin tables, MSKCCn, and CAPRA score. In our study, the diagnostic efficacy of the clinical model was also significantly improved, from (0.50, 0.78) to (0.79, 0.83), respectively, after the EPE classification combined with the Partin tables and the MSKCCn and the combined model was obviously able to obtain higher clinical benefits. The result was similar to the study by Xu et al. [13] Therefore, compared with the clinical model alone, the addition of EPE grade can improve the accuracy of clinical evaluation of prostate cancer and help clinicians to develop personalized treatment plans. Because the neurovascular bundle cannot be preserved, most patients develop urinary incontinence and sexual dysfunction after surgery [17], which significantly reduces the patient's quality of life and increases the social burden. The limitations of this study are as follows: (1) to assess the role of EPE grade in personalized decision making, a prospective multicenter study is required. (2) The relationship between EPE grade and incision state and lymph node invasion was not analyzed, and further research is needed to explore. (3) Without the use of a prostatic rectal coil, it may be that the imaging assessment of EPE with a rectal coil will have different results, which requires further study.

## 5. Conclusion

In summary, EPE grade is a simple and standardized grading method, which has high accuracy in predicting EPE of prostate cancer. And it can significantly improve the diagnostic efficiency of clinical models and increase clinical benefits after combining with clinical models.

## Figures and Tables

**Table 1:** Clinicopathological characteristics of patients in this study (*n* = 105).

Variable	Value
Age (y)^∗^	69.2 ± 5.8 (52-82)
Prostate-specific antigen (mg/ml)^∗^	23.4 ± 24.0 (3.1-141.9)
Percentage of positive biopsy cores^∗^	0.45 ± 0.27 (0.08-1)
ISUP category at biopsy	

1	13

2	16

3	27

4	21

5	28
cT stage	

1	76

2a	7

2b/2c	22
EPE grade	

0	70

1	18

2	10

3	7
Pathologic EPE	

Present	44

Absent	61

ISUP: International Society of Urological Pathology. ^∗^Data are the mean ± standard deviation (range).

**Table 2:** The AUC value of sensitivity and specificity in EPE prediction schemes.

Prediction schemes	AUC value (95% CI)	Sensibility	Specificity
EPE grade	0.79 (0.702-0.865)	68%	90%
Partin tables	0.50 (0.404-0.602)	82%	34%
MSKCCn	0.78 (0.690-0.856)	71%	79%
EPE grade+Partin tables	0.79 (0.699-0.863)	68%	90%
EPE grade+MSKCCn	0.83 (0.748-0.899)	73%	87%

**Table 3:** Comparison of AUC values in different EPE prediction schemes.

	EPE grade	Partin tables	MSKCCn	EPE grade+Partin tables	EPE grade+MSKCCn
EPE grade	—	—	—	—	—
Partin tables	*P* < 0.001	—	—	—	—
MSKCCn	*P* = 0.803	*P* < 0.001	—	—	—
EPE grade+Partin tables	*P* = 0.936	*P* < 0.001	*P* = 0.856	—	—
EPE grade+MSKCCn	*P* = 0.116	*P* < 0.001	*P* = 0.024	*P* = 0.211	—

## Data Availability

All the data relevant to this study are mentioned in the manuscript.
